# Development and Evaluation of Novel and Highly Sensitive Single-Tube Nested Real-Time RT-PCR Assays for SARS-CoV-2 Detection

**DOI:** 10.3390/ijms21165674

**Published:** 2020-08-07

**Authors:** Cyril Chik-Yan Yip, Siddharth Sridhar, Kit-Hang Leung, Anthony Chin-Ki Ng, Kwok-Hung Chan, Jasper Fuk-Woo Chan, Owen Tak-Yin Tsang, Ivan Fan-Ngai Hung, Vincent Chi-Chung Cheng, Kwok-Yung Yuen, Kelvin Kai-Wang To

**Affiliations:** 1 Department of Microbiology, Queen Mary Hospital, Hong Kong, China; yipcyril@hku.hk (C.C.-Y.Y.); vcccheng@hku.hk (V.C.-C.C.); 2 Department of Microbiology, The University of Hong Kong, Hong Kong, China; sid8998@hku.hk (S.S.); khl17@hku.hk (K.-H.L.); anthonyng912@gmail.com (A.C.-K.N.); chankh2@hku.hk (K.-H.C.); jfwchan@hku.hk (J.F.-W.C.); 3 State Key Laboratory of Emerging Infectious Diseases, The University of Hong Kong, Hong Kong, China; 4 Carol Yu Centre for Infection, Li Ka Shing Faculty of Medicine, The University of Hong Kong, Hong Kong, China; 5 Department of Medicine and Geriatrics, Princess Margaret Hospital, Hong Kong, China; tsangty@ha.org.hk; 6 Department of Medicine, The University of Hong Kong, Hong Kong, China; ivanhung@hku.hk

**Keywords:** SARS-CoV-2, COVID-19, single-tube nested, RT-PCR, evaluation

## Abstract

Sensitive molecular assays are critical for coronavirus disease 2019 (COVID-19) diagnosis. Here, we designed and evaluated two single-tube nested (STN) real-time RT-PCR assays, targeting SARS-CoV-2 RdRp/Hel and *N* genes. Both STN assays had a low limit of detection and did not cross react with other human coronaviruses and respiratory viruses. Using 213 initial respiratory specimens from suspected COVID-19 patients, the sensitivity of both the STN COVID-19-RdRp/Hel and the STN COVID-19-N assays was 100% (99/99), while that of the comparator non-nested *N* assay was 95% (94/99). Among 108 follow-up specimens from confirmed COVID-19 patients who tested negative by the non-nested COVID-19-RdRp/Hel assay, 28 (25.9%) were positive for SARS-CoV-2 by the STN COVID-19-RdRp/Hel or the STN COVID-19-N assay. To evaluate the performance of our novel STN assays in pooled specimens, we created four sample pools, with each pool consisting of one low positive specimen and 49 negative specimens. While the non-nested COVID-19-RdRp/Hel assay was positive in only one of four sample pools (25%), both of the STN assays were positive in two of four samples pools (50%). In conclusion, the STN assays are highly sensitive and specific for SARS-CoV-2 detection. Their boosted sensitivity offers advantages in non-traditional COVID-19 testing algorithms such as saliva screening and pooled sample screening during massive screening.

## 1. Introduction

The global pandemic of coronavirus disease 2019 (COVID-19) is the greatest infectious disease challenge of the 21st century [1,2,3,4]. Various non-pharmaceutical public health interventions to reduce COVID-19 community transmission have been instituted worldwide [5,6,7]. Widespread deployment of accessible and accurate COVID-19 RT-PCR tests for individuals with mild symptoms in the community is an important way to reduce COVID-19 transmission. Confirmed cases of COVID-19 may then be isolated and their close contacts quarantined to dramatically reduce the effective reproduction number [8].

There is evidence to suggest that viral loads of SARS-CoV-2, the causative agent of COVID-19, are higher in lower respiratory tract specimens than upper respiratory tract specimens [9]. However, the usual specimen types obtained from patients with suspected COVID-19 are nasopharyngeal and oropharyngeal swabs [10,11]. Poor sampling techniques can further affect the accuracy of tests run on these specimens. These factors raise the possibility of “false-negative” results from upper respiratory tract specimens. One approach to overcome this shortcoming is to maximize the analytical sensitivity of the COVID-19 reverse transcription-polymerase chain reaction (RT-PCR) assay so as to be able to detect even minute levels of SARS-CoV-2 RNA in upper respiratory tract specimens.

Nested PCR assays utilize two sets of primers (external and internal) to maximize analytical sensitivity, but the major drawback of these assays is the need to transfer amplicons from the external primer reaction to a new tube for the internal primer reaction. Furthermore, traditional nested assays rely on end-point product detection in gels and so are not amenable to quantitation. Single-tube nested (STN) real-time RT-PCR assays overcome these limitations by manipulating annealing temperatures, thereby enabling reverse transcription, external primer amplification and internal primer amplification to take place sequentially in the same tube [12]. By incorporation of fluorescent probes, these assays are also compatible with commonly used real-time PCR thermocyclers enabling relative quantitation. Recently, Wang et al. has developed SARS-CoV-2 STN RT-PCR assays based on a commercial RT-PCR kit [13], but the primer and probe sequences used were not reported. In this study, we designed novel STN real-time RT-PCR assays capable of highly sensitive detection of SARS-CoV-2 in clinical specimens.

## 2. Results

### 2.1. In Silico Analysis of Primers and Probes Used in the In-House Developed STN Real-Time RT-PCR Assays for SARS-CoV-2 Detection

To assess whether the novel STN real-time RT-PCR assays can detect globally circulating SARS-CoV-2 isolates, the primer and probe sequences were aligned with viral gene sequences that are available in NCBI GenBank (Table S1). The primers and probes showed no mismatches with SARS-CoV-2 isolates from different geographical regions, except one noted at the 5′ end of the inner reverse primer of the COVID-19-RdRp/Hel assay (Figure S1). These primer/probe sets were predicted to specifically detect SARS-CoV-2 and had no major combined homologies with human, other respiratory viruses or microbial genes on BLASTn analysis that would potentially produce false-positive test results.

### 2.2. Analytical Performance of the STN RT-PCR Assays for SARS-CoV-2 Detection

To determine the analytical sensitivity of the STN RT-PCR assays, the limit of detection (LOD) was evaluated by using total nucleic acid (TNA) extracted from the SARS-CoV-2 isolate. Serial 10-fold dilutions of SARS-CoV-2 TNA extracted from the viral culture isolate were prepared and tested in triplicate for each concentration in two independent runs. The LOD of both the STN COVID-19-RdRp/Hel assay and the STN COVID-19-N assay was 1.8 × 10^−1^ TCID_50_/mL (Table S2).

To determine the analytical specificity of the STN COVID-19-RdRp/Hel assay and the STN COVID-19-N real-time RT-PCR assays, we tested TNA extracted from a clinical respiratory specimen with human coronavirus HKU1 (HCoV-HKU1), and TNAs extracted from the 17 culture isolates of SARS-CoV, MERS-CoV, HCoV-OC43, HCoV-NL63, HCoV-229E, influenza A ((H1N1)pdm09 and H3N2) viruses, influenza B virus, influenza C virus, parainfluenza virus types 1–4, respiratory syncytial virus, human metapneumovirus, human rhinovirus and human adenovirus. None of the two STN assays cross reacted with other respiratory viruses, confirming that they were highly specific.

### 2.3. Diagnostic Performance of the STN RT-PCR Assays for SARS-CoV-2 Detection

**Figure 1:**
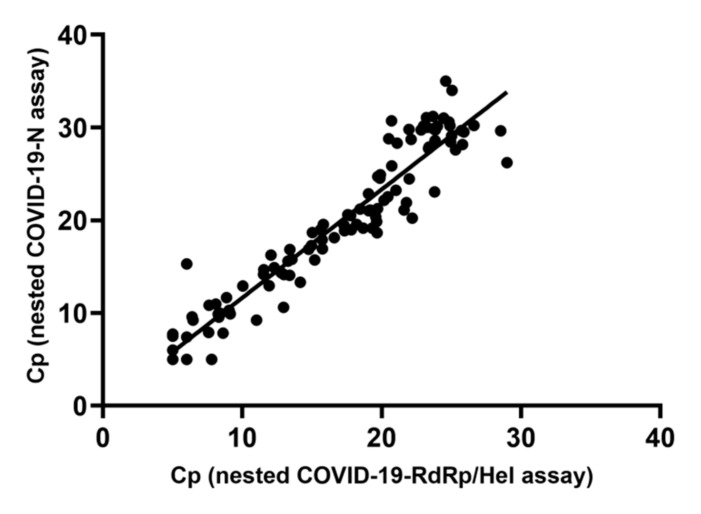
Correlation of the Cp values of the specimens found positive for SARS-CoV-2 by the STN COVID-19-RdRp/Hel assay and the STN COVID-19-N assay.

**Figure 2:**
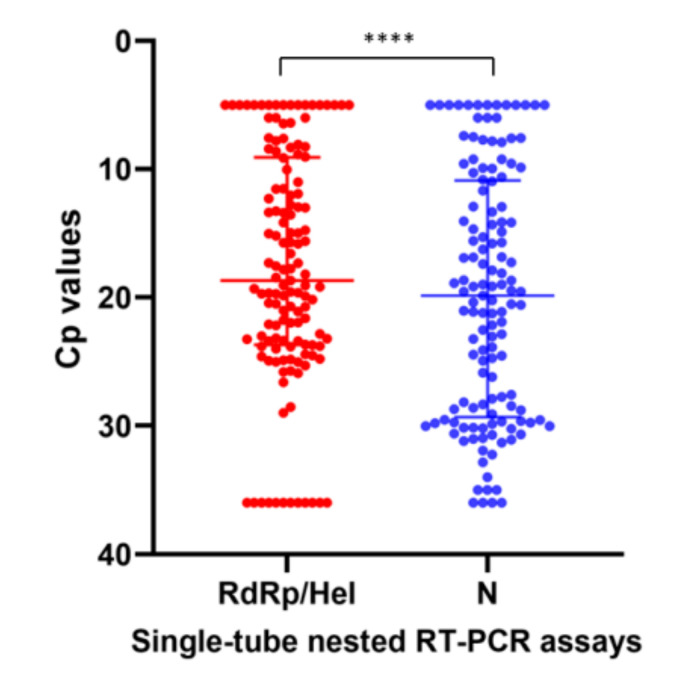
Comparison of the Cp values of the two STN RT-PCR assays in this study. A Cp value of 36 was assigned to specimens that tested negative in the STN RT-PCR assays. **** indicates *p* < 0.0001.

To assess the diagnostic performance of the STN real-time RT-PCR assays in clinical specimens, a total of 213 initial respiratory specimens from suspected COVID-19 patients were subjected to SARS-CoV-2 detection by the in-house developed STN COVID-19-RdRp/Hel and STN COVID-19-N RT-PCR assays, the non-nested COVID-19-RdRp/Hel RT-PCR assay and the non-nested RT-PCR assay targeting the *N* gene designed by National Institute of Infectious Diseases (NIID), Japan. Among these 213 specimens, 99 were positive for SARS-CoV-2 by the two STN RT-PCR assays and the non-nested COVID-19-RdRp/Hel assay, while 94 were positive for SARS-CoV-2 by the Japanese NIID non-nested *N* assay (Table 1). For the five specimens that tested negative by the non-nested *N* assay but positive by the other three assays, the median Cp value of these specimens was 36.13 (range: 34.80–37.61) by the non-nested COVID-19-RdRp/Hel assay, which represented low viral load. Among the eight proficiency testing (PT) samples from Quality Control for Molecular Diagnostics (QCMD), the two STN RT-PCR assays gave 100% correct results. A good agreement between Cp values generated by the STN COVID-19-RdRp/Hel and STN COVID-19-N RT-PCR assays was shown by a strong correlation (Spearman’s ρ = 0.96; *p* < 0.0001) (Figure 1). The Cp values obtained from the two STN RT-PCR assays were also examined. The median Cp value of the STN COVID-19-RdRp/Hel RT-PCR assay (18.67, IQR 9.09–23.69) was significantly lower than that of the STN COVID-19-N RT-PCR assay (19.85, IQR 10.89–29.32) (*p* < 0.0001) (Figure 2).

To further determine the clinical utility of the STN RT-PCR assays, 91 follow-up respiratory specimens and 17 follow-up non-respiratory specimens from patients with laboratory-confirmed COVID-19, which tested negative for SARS-CoV-2 by the non-nested COVID-19-RdRp/Hel assay, were tested. These specimens were collected from the patients with COVID-19 between 2 and 42 days after onset of symptoms. Among the 91 respiratory specimens, 21 (23.1%) were positive by either the STN COVID-19-RdRp/Hel assay or the STN COVID-19-N assays, of which 9 (9.9%) were positive by both assays (Table S3). Among the 17 non-respiratory specimens, 7 (41.2%) were positive by either the STN COVID-19-RdRp/Hel assay or the STN COVID-19-N assays, of which 3 (17.6%) were positive by both assays (Table S3).

Simultaneously collected nasopharyngeal swab (NPS) and saliva specimens obtained from eight COVID-19 patients were available for testing. One saliva specimen (Patient H) tested negative by the non-nested COVID-19-RdRp/Hel assay was positive for SARS-CoV-2 by the two STN RT-PCR assays (Table S4).

To determine whether a positive sample with a low viral load (Cp > 31) spiked in a pool of 50 samples could be detected by the STN RT-PCR assays, 4 pooled samples (50 samples per pool) were tested (Table 2). Each pool consisted of one positive sample with high Cp values combined with 49 SARS-CoV-2-negative samples. When the Cp value of the positive sample was approximately 32, both the non-nested COVID-19-RdRp/Hel assay and the STN RT-PCR assays flagged the pool positive. However, when the positive sample had a Cp value of approximately 33, the two STN RT-PCR assays, but not the non-nested COVID-19-RdRp/Hel assay, detected SARS-CoV-2 RNA in the pool. For the pools containing a positive sample with Cp > 34, the non-nested and STN RT-PCR assays flagged the pools as negative.

## 3. Discussion

RT-PCR assays are the most widely performed diagnostic tests for COVID-19 (https://www.who.int/publications/m/item/molecular-assays-to-diagnose-covid-19-summary-table-of-available-protocols). Despite their reliability and impressive analytical sensitivity, their real-world diagnostic sensitivity is often limited by sampling from sites with lower viral loads or using incorrect collection techniques. In this study, we present a potential solution to this problem by maximizing the analytical sensitivity of the assay with a nested real-time RT-PCR approach. This design combines the convenience of real-time RT-PCR with the high specificity and sensitivity of nested PCR.

Our results in this study offer a proof of concept of this approach. The analytical sensitivity of the STN RT-PCR assays targeting the RNA-dependent RNA polymerase (RdRp)/Helicase (Hel) and nucleocapsid (*N*) genes was higher than that of their non-nested counterparts using the same inner primers. The LOD of the two STN assays was one log_10_ lower than that of the non-nested assays (1.8 TCID_50_/mL, which was determined in our previous study) [14]. The diagnostic sensitivity of the STN RT-PCR assays for initial clinical specimens from suspected COVID-19 patients was equivalent to the non-nested RdRp/Hel RT-PCR and superior to the Japanese NIID *N* gene RT-PCR assay. However, when we assessed follow-up specimens from COVID-19 patients containing declining viral loads, a clear pattern of the superior diagnostic sensitivity of the STN RT-PCR assays was observed. This suggests that the additional analytical sensitivity of these assays translated to higher diagnostic sensitivities when deployed in specimens with low viral load. The STN RT-PCR assays also enabled us to diagnose COVID-19 from posterior oropharyngeal saliva, which is much more economical and safe to collect compared to swab specimens [16,17].

Rapid deployment of COVID-19 testing has been one of the major hurdles to the pandemic response in countries around the world. The cost of testing is often prohibitive in resource-limited settings. Moreover, there is a possibility of the shortage of nucleic acid extraction kits due to the heavy demand on SARS-CoV-2 screening during the COVID-19 outbreaks. One of the proposed solutions has been to pool respiratory samples together for testing, followed by work up of individual samples only if the pool is positive [18]. Pooling samples reduces the analyte load and, therefore, using highly sensitive nested RT-PCR assays offers obvious advantages. In our study, we found that the two STN RT-PCR assays offered additional sensitivity over the non-nested assay when the Cp value of the positive sample in the pool was approximately 33. This finding was similar to a study showing that SARS-CoV-2 RNA could still be detected in the positive pools with the single samples having the threshold cycle value up to 34, in which each pool consisted of 30 samples [18].

To date, primers and probes used in different RT-PCR assays mainly target *ORF1ab*, *S*, *E*, *N* regions of SARS-CoV-2 [1,14,17,19,20,21,22,23,24]. Coronaviruses have a high frequency of mutations and recombination [25,26,27], which may result in mismatches with the currently used primers and probes. Based on the multiple sequence alignment we performed in this study, no mismatch was noted between the primers/probes we used in the present study and the corresponding gene regions of the SARS-CoV-2 isolates circulating globally, except one noted at the 5′ end of the inner reverse primer of the COVID-19-RdRp/Hel assay. The mismatch was intentionally made to achieve the optimal GC content and melting temperature of this primer. The position of this mismatch is unlikely to affect the sensitivity of the assay, as shown in our evaluation. However, as these assays involve an additional set of primers, regular in silico surveillance to monitor for escape variants is required.

There are several differences between our SARS-CoV-2 STN RT-PCR assays and those reported by Wang et al. [13]. First, unlike our assays, these assays required locked nucleic acids in their external primer design and their assays with internal primer sets were commercial ones, which would dramatically increase assay cost. Second, their assay also required a relatively large template volume of 20 µL, while we achieved equivalent sensitivity with a smaller template volume of 5 µL.

Although STN RT-PCR assays represent a significant improvement over traditional real-time RT-PCR assays, their high sensitivity could be a disadvantage. As infection control regulations in many countries require negative SARS-CoV-2 RT-PCR results prior to patient discharge, adopting highly sensitive RT-PCR assays would be a disadvantage due to their enhanced ability to detect non-viable viral RNA fragments. We believe their role is mainly in initial screening of clinical specimens or pooled screening, as discussed above.

A limitation of this study was that we did not perform cell culture for STN RT-PCR positive, non-nested RT-PCR-negative specimens to investigate the viability of the virus. It is also probable that the efficacy of nucleic acid extraction is a limiting factor to the diagnostic sensitivity of STN RT-PCR assays. Future studies should compare nested and non-nested RT-PCR assays using different extraction methods. The relative importance of different gene targets and the optimal target for STN RT-PCR assays also remains to be elucidated.

In conclusion, this study describes the design and evaluation of novel STN RT-PCR assays for COVID-19 diagnosis. These assays can be applied to non-traditional specimen types such as posterior throat saliva. They maximize the sensitivity of initial screening of clinical specimens from patients with suspected COVID-19.

## 4. Materials and Methods 

### 4.1. Viruses, Clinical Specimens and Proficiency Testing Samples for Evaluation

SARS-CoV-2 *HKU-001a* (GenBank accession number MT230904) was isolated from the nasopharyngeal aspirate (NPA) of a patient with COVID-19 in Hong Kong [28]. A SARS-CoV-2 culture isolate stock (1.8 × 10^7^ TCID_50_/mL) was prepared by one additional passage using VeroE6 cell lines [29,30]. For analytical sensitivity evaluation, 10-fold serial dilutions of TNA extracted from the SARS-CoV-2 culture isolate were used. For analytical specificity evaluation, TNA extracted from a clinical specimen positive for HCoV-HKU1 and 17 culture isolates of other human coronaviruses and respiratory viruses were used [19,20,31]. For diagnostic performance evaluation, 213 initial clinical specimens including NPA, NPS, throat swab or saliva collected from 213 hospitalized patients (male: female = 112: 101; median age: 48 years; range: 62 days–103 years) with suspected COVID-19 were included for SARS-CoV-2 detection. Some of these specimens were evaluated previously using our in-house RT-PCR assays for SARS-CoV-2 detection [21]. To further determine the clinical utility of our novel STN real-time RT-PCR assays, we included hospitalized patients with sequential respiratory and non-respiratory specimens sent for SARS-CoV-2 detection for whom the first specimen tested positive but subsequent specimens tested negative by our previously described validated COVID-19-RdRp/Hel real-time RT-PCR assay [14]. We also included patients who had discrepant COVID-19-RdRp/Hel RT-PCR assay results on NPS and saliva specimens collected on the same day. These specimens were evaluated by a point-of-care assay in our previous study [32]. Furthermore, we prepared four pooled samples, with each pool consisting of one SARS-CoV-2-positive specimen with low viral load and 49 SARS-CoV-2-negative specimens. These pooled samples were evaluated by the non-nested COVID-19-RdRp/Hel assay and the STN real-time RT-PCR assays. In addition to clinical specimens, eight PT samples from QCMD with various concentrations of SARS-CoV-2 or negative for SARS-CoV-2 were also evaluated. The present study has been approved by the Institutional Review Board of the University of Hong Kong/Hospital Authority Hong Kong West Cluster (UW 20–286).

### 4.2. Nucleic Acid Extraction

TNA extraction of clinical specimens, pooled samples, PT samples and virus culture isolates was performed on 250 μL of each sample using the NucliSENS easyMAG extraction system (bioMérieux, Marcy-l’Étoile, France), as we previously described [33]. The TNA extracts (each with 55 μL) were stored at −80 °C until use.

### 4.3. Primers and Probes

Primer (outer and inner) and probe sets targeting the RdRp/Hel and *N* gene regions of SARS-CoV-2 for in-house developed STN RT-PCR assays were designed and tested. Multiple sequence alignments using the primer/probe sequences and the gene sequences of global SARS-CoV-2 isolates downloaded from NCBI GenBank were performed using ClustalX 2.1 [34].

### 4.4. Real-Time RT-PCR Assays for SARS-CoV-2 RNA Detection

A non-nested COVID-19-RdRp/Hel real-time RT-PCR assay for SARS-CoV-2 RNA detection was performed using a QuantiNova Probe RT-PCR Kit (QIAGEN, Hilden, Germany), as we previously described [14]. Each 20 μL reaction mixture contained 10 μL of 2× QuantiNova Probe RT-PCR Master Mix, 0.2 μL of QN Probe RT-Mix, 1.6 μL of each 10 μM forward and reverse primer, 0.4 μL of 10 μM probe (Table 3), 1.2 μL of nuclease-free water and 5 μL of TNA. Amplification and detection were performed on a LightCycler 480 II Real-Time PCR System (Roche, Basel, Switzerland). Thermocycling conditions consisted of 10 min at 45 °C for reverse transcription, 5 min at 95 °C for PCR initial activation, and 45 cycles of 5 s at 95 °C and 30 s at 55 °C.

One of the in-house developed molecular assays listed on the World Health Organization (WHO) website (https://www.who.int/who-documents-detail/molecular-assays-to-diagnose-covid-19-summary-table-of-available-protocols) was also used for comparative evaluation in this study. The non-nested *N* assay was designed by NIID, Japan [15], and we used the same protocol listed on the WHO website (https://www.who.int/docs/default-source/coronaviruse/whoinhouseassays.pdf?sfvrsn=de3a76aa_2&download=true). Briefly, a one-step real-time RT-PCR was performed using QuantiTect Probe RT-PCR Kit (QIAGEN) on the LightCycler 96 real-time PCR system (Roche). Each 20 μL reaction mixture contained 10 μL of 2× Master Mix, 0.2 μL of RT mix, 1 μL of 10 μM forward primer, 1.4 μL of 10 μM reverse primer, 0.4 μL of 10 μM probe (Table 3), 2 μL of nuclease-free water and 5 μL of TNA. Thermocycling conditions consisted of 30 min at 50 °C and 15 min at 95 °C, followed by 40 cycles of 15 s at 95 °C and 1 min at 60 °C.

The STN real-time RT-PCR assays were performed using the QuantiNova Probe RT-PCR Kit (QIAGEN) on the LightCycler 480 II Real-Time PCR System (Roche). Each 20 μL reaction mixture contained 10 μL of 2× QuantiNova Probe RT-PCR Master Mix, 0.2 μL of QN Probe RT-Mix, 0.16 μL of each 10 μM outer forward and reverse primer, 1.6 μL of each 10 μM inner forward and reverse primer, 0.4 μL of 10 μM probe (Table 3), 0.88 μL of nuclease-free water and 5 μL of TNA as the template. Thermocycling conditions consisted of 10 min at 45 °C for reverse transcription, 5 min at 95 °C for PCR initial activation, followed by 20 cycles of 5 s at 95 °C and 30 s at 69 °C (for first-round amplification by external primers with higher melting temperature (Tm)), and then 40 cycles of 5 s at 95 °C and 30 s at 55 °C (for second-round amplification by inner primers with a lower Tm; with fluorescence signal detection) (Figure S2). Negative and positive controls were included in all runs to monitor assay performance. PCR data were analyzed by “Abs Quant/2nd Derivative Max” method using the LightCycler 480 II system, which generated a Cp value of 5 for the samples with a high viral load between 0 and 5 cycles under default settings.

### 4.5. Statistical Analysis

Kappa statistic was used to determine the agreement between an assay and the reference standard (results obtained from at least 3 of 4 assays). McNemar’s test was used to compare the performance of the assays with the reference standard. Spearman’s correlation was used to assess the relation between the Cp values of the two STN real-time RT-PCR assays. Cp values of the two STN real-time RT-PCR assays were compared using Wilcoxon signed-rank test (a Cp value of 36 was assigned to specimens that tested negative in the STN RT-PCR assays). *p* < 0.05 was considered statistically significant. All statistical analysis was performed using GraphPad PRISM 8 or SPSS 26.0.

## Figures and Tables

**Table 1:** Clinical performance comparison of the in-house developed STN real-time RT-PCR assays and other validated molecular assays for SARS-CoV-2 detection.

Molecular Assays		Reference Standard *		Kappa Value (95% CI) ^†^	McNemar’s Test
		Positive	Negative		
STN COVID-19-RdRp/Hel assay					
	Positive	99	0	1.00 (1.00–1.00)	*p* = 1.000
	Negative	0	114		
STN COVID-19-N assay					
	Positive	99	0	1.00 (1.00–1.00)	*p* = 1.000
	Negative	0	114		
Non-nested COVID-19-RdRp/Hel assay [14]					
	Positive	99	0	1.00 (1.00–1.00)	*p* = 1.000
	Negative	0	114		
Non-nested *N* assay [15]					
	Positive	94	0	0.95 (0.91–0.99)	*p* = 0.063
	Negative	5	114		

* The reference standard was defined as the results obtained from at least three of the above assays. **^†^** CI, confidence interval.

**Table 2:** Evaluation of four pooled samples using the in-house developed non-nested COVID-19-RdRp/Hel and STN real-time RT-PCR assays.

	Cp Value			
Pool *	Single Test (Non-Nested COVID-19-RdRp/Hel Assay)	Pool Test (Non-Nested COVID-19-RdRp/Hel Assay	Pool Test (STN COVID-19-RdRp/Hel Assay)	Pool Test (STN COVID-19-N Assay)
1	32.21	32.62	21.03	24.68
2	33.31	−	23.25	30.25
3	34.80	−	−	−
4	35.76	−	−	−

−, negative. * 50 samples per pool (each pool with one positive sample and 49 negative samples by the non-nested COVID-19-RdRp/Hel assay).

**Table 3:** Primers and probes used in this study.

Primer/Probe	Sequence (5′–3′)	Gene Target	Reference
**In-house single-tube nested real-time RT-PCR**			
Outer forward	AGGTATTGGGAACCTGAGTTTTATGAGGCTATGTACACAC	RdRp/Hel	This study
Outer reverse	ACCTGGAGCATTGCAAACATACGGATTAACAGACAAGAC		
Inner forward	CGCATACAGTCTTRCAGGCT		
Inner reverse	GTGTGATGTTGAWATGACATGGTC		
Probe	FAM- TTAAGATGTGGTGCTTGCATACGTAGAC -lABkFQ		
Outer forward	AATTGCACAATTTGCCCCCAGCGCTTCA	N	This study
Outer reverse	TGCGTCAATATGCTTATTCAGCAAAATGACTTGATCTTTGA		
Inner forward	GCGTTCTTCGGAATGTCG		
Inner reverse	TTGGATCTTTGTCATCCAATTTG		
Probe	FAM- AACGTGGTTGACCTACACAGST -lABkFQ		
**In-house non-nested real-time RT-PCR**			
COVID-19-RdRp/Hel-F	CGCATACAGTCTTRCAGGCT	RdRp/Hel	[14]
COVID-19-RdRp/Hel-R	GTGTGATGTTGAWATGACATGGTC		
COVID-19-RdRp/Hel-P	FAM- TTAAGATGTGGTGCTTGCATACGTAGAC -lABkFQ		
NIID_2019-n COV_N_F2	AAATTTTGGGGACCAGGAAC	N	[15]
NIID_2019-n COV_N_R2	TGGCAGCTGTGTAGGTCAAC		
NIID_2019-n COV_N_P2	FAM- ATGTCGCGCATTGGCATGGA -BHQ		

## Supplementary Materials

Supplementary materials can be found at https://www.mdpi.com/1422-0067/21/16/5674/s1.

## Abbreviations

BLASTn	Nucleotide Basic Local Alignment Search Tool
COVID-19	Coronavirus disease 2019
HCoV	Human coronavirus
Hel	Helicase
LOD	Limit of detection
MERS-CoV	Middle East respiratory syndrome coronavirus
N	Nucleocapsid
NCBI	National Center for Biotechnology Information, USA
NPA	Nasopharyngeal aspirate
NPS	Nasopharyngeal swab
PT	Proficiency testing
QCMD	Quality Control for Molecular Diagnostics
RdRp	RNA-dependent RNA polymerase
RT-PCR	Reverse transcription-polymerase chain reaction
SARS-CoV	Severe acute respiratory syndrome coronavirus
SARS-CoV-2	Severe acute respiratory syndrome coronavirus 2
STN	Single-tube nested
TCID_50_	Tissue culture infectious dose
Tm	Melting temperature
TNA	Total nucleic acid
WHO	World Health Organization
