# Supplementary material for: Development and Evaluation of Novel and Highly Sensitive Single-Tube Nested Real-Time RT-PCR Assays for SARS-CoV-2 Detection
Source: Int J Mol Sci. 2020 Aug 7;21(16):5674. doi: 10.3390/ijms21165674 (PMC7461039; doi:10.3390/ijms21165674)
Supplement: Supplementary file 1 [file ijms-21-05674-s001.pdf]

Table S1. List of SARS-CoV-2 isolates used in the multiple sequence alignment focused on primer/probe binding sites

Sequence number	Isolate name	Collection date	Country
**GenBank**			
MN908947.3	Wuhan-Hu-1	Dec-2019	China
MT019529.1	BetaCoV/Wuhan/IPBCAMS-WH-01/2019	23-Dec-2019	China
MN996527.1	WIV02	30-Dec-2019	China
MN988668.1	2019-nCoV WHU01	02-Jan-2020	China
MN938384.1	2019-nCoV_HKU-SZ-002a_2020	10-Jan-2020	China
MN975262.1	2019-nCoV_HKU-SZ-005b_2020	11-Jan-2020	China
MT039873.1	HZ-1	20-Jan-2020	China
MT121215.1	SARS-CoV-2/human/CHN/SH01/2020	02-Feb-2020	China
LC528232.1	SARS-CoV-2/Hu/DP/Kng/19-020	10-Feb-2020	Japan
MT304475.1	SARS-CoV-2/human/KOR/BA-ACH_2718/2020	29-Feb-2020	South Korea
MT066175.1	SARS-CoV-2/NTU01/TWN/human/2020	31-Jan-2020	Taiwan
MT192772.1	SARS-CoV-2/human/VNM/nCoV-19-01S/2020	22-Jan-2020	Vietnam
MT072688.1	SARS-CoV-2/human/NPL/61-TW/2020	13-Jan-2020	Nepal
MT276598.1	SARS-CoV-2/human/ISR/ISR_IT0320/2020	Mar-2020	Israel
MT262993.1	SARS-Cov-2/human/PAK/Manga1/2020	12-Mar-2020	Pakistan
MT012098.1	SARS-CoV-2/human/IND/29/2020	27-Jan-2020	India
MT007544.1	Australia/VIC01/2020	25-Jan-2020	Australia
MT451786.1	SARS-CoV-2/human/AUS/VIC1178/2020	10-Apr-2020	Australia
MN985325.1	2019-nCoV/USA-WA1/2020	19-Jan-2020	USA
MT039888.1	2019-nCoV/USA-MA1/2020	29-Jan-2020	USA
MT106054.1	2019-nCoV/USA-TX1/2020	11-Feb-2020	USA
MT159717.1	2019-nCoV/USA-CruiseA-1/2020	17-Feb-2020	USA
MT152824.1	SARS-CoV-2/human/USA/WA2/2020	24-Feb-2020	USA
MT246461.1	SARS-CoV-2/human/USA/WA-UW204/2020	13-Mar-2020	USA
MT126808.1	SARS-CoV-2/human/BRA/SP02/2020	28-Feb-2020	Brazil
MT256924.1	SARS-CoV-2/human/COL/79256_Antioquia/2020	11-Mar-2020	Colombia
MT263074.1	SARS-CoV-2/human/PER/Peru-10/2020	10-Mar-2020	Peru
MT020781.2	nCoV-FIN-29-Jan-2020	29-Jan-2020	Finland
MT093571.1	SARS-CoV-2/human/SWE/01/2020	07-Feb-2020	Sweden
MT066156.1	SARS-CoV-2/human/ITA/INMI1/2020	30-Jan-2020	Italy
MT198652.2	SARS-CoV-2/human/ESP/Valencia003/2020	05-Mar-2020	Spain
MT470179.1	SARS-CoV-2/human/France/40002VJ/2020	Mar-2020	France
MT358643.1	SARS-CoV-2/human/DEU/FFM7/2020	Feb-2020	Germany
MT324062.1	SARS-CoV-2/human/ZAF/R03006/2020	07-Mar-2020	South Africa

Table S2. Test results for determining the limit of detection of the in-house developed single-tube nested (STN) real-time RT-PCR assays with genomic RNA extracted from a SARS-CoV-2 culture isolate

**Virus titer ($TCID_{50}$/mL)**	**Cp (Intra-run)**			**Cp (Inter-run)**		
	Test 1	Test 2	Test 3	Test 1	Test 2	Test 3
STN COVID-19-RdRp/Hel assay						
1.8×10^{1}	20.79	20.40	20.86	19.46	19.25	19.19
1.8×10^{0}	24.34	23.52	25.27	22.56	21.45	21.98
1.8×10^{-1}	26.17	25.76	23.96	25.34	23.99	25.18
1.8×10^{-2}	28.37	-	-	-	-	27.49
1.8×10^{-3}	37.87	-	-	-	27.62	-
STN COVID-19-N assay						
1.8×10^{1}	21.91	19.86	22.53	19.59	19.80	19.08
1.8×10^{0}	27.69	22.17	25.25	22.67	24.01	24.11
1.8×10^{-1}	28.75	31.26	29.19	27.99	27.56	28.80
1.8×10^{-2}	-	-	-	30.29	-	29.91
1.8×10^{-3}	-	-	-	-	-	-

Table S3. Performance of the STN COVID-19-RdRp/Hel and STN COVID-19-N assays on follow-up specimens from COVID-19 patients that tested negative by non-nested COVID-19-RdRp/Hel assay

**Respiratory specimens (NPA/NPS/TS/saliva)**	No. of positive specimens (%) (n=91)
Positive by both STN assays	9 (9.9)
Positive by STN COVID-19-RdRp/Hel assay only	3 (3.3)
Positive by STN COVID-19-N assay only	9 (9.9)

NPA, nasopharyngeal aspirate; NPS, nasopharyngeal swab; TS, throat swab

**Non-respiratory specimens (rectal swabs/stool/plasma)**	No. of positive specimens (%) (n=17)
Positive by both STN assays	3 (17.6)
Positive by STN COVID-19-RdRp/Hel assay only	1 (5.9)
Positive by STN COVID-19-N assay only	3 (17.6)

Table S4. Evaluation of the paired NPS and saliva specimens from the confirmed COVID-19 cases with discrepant results by the non-nested COVID-19-RdRp/Hel assay using the STN real-time RT-PCR assays

		Cp value		
**Patient**	**Specimen**	**Non-nested COVID-19-RdRp/Hel assay**	**STN COVID-19-RdRp/Hel assay**	**STN COVID-19-N assay**
A	NPS	35.20	20.64	20.67
	Saliva	-	-	-
B	NPS	-	-	-
	Saliva	28.80	17.33	17.91
C	NPS	-	-	-
	Saliva	23.10	13.83	14.36
D	NPS	32.31	19.90	21.04
	Saliva	-	-	-
E	NPS	36.96	28.84	31.22
	Saliva	-	-	-
F	NPS	-	-	-
	Saliva	28.10	18.38	16.81
G	NPS	36.26	23.21	31.08
	Saliva	-	-	-
H	NPS	35.37	22.10	28.71
	Saliva	-	22.18	20.23

-, negative; NPS, nasopharyngeal swab

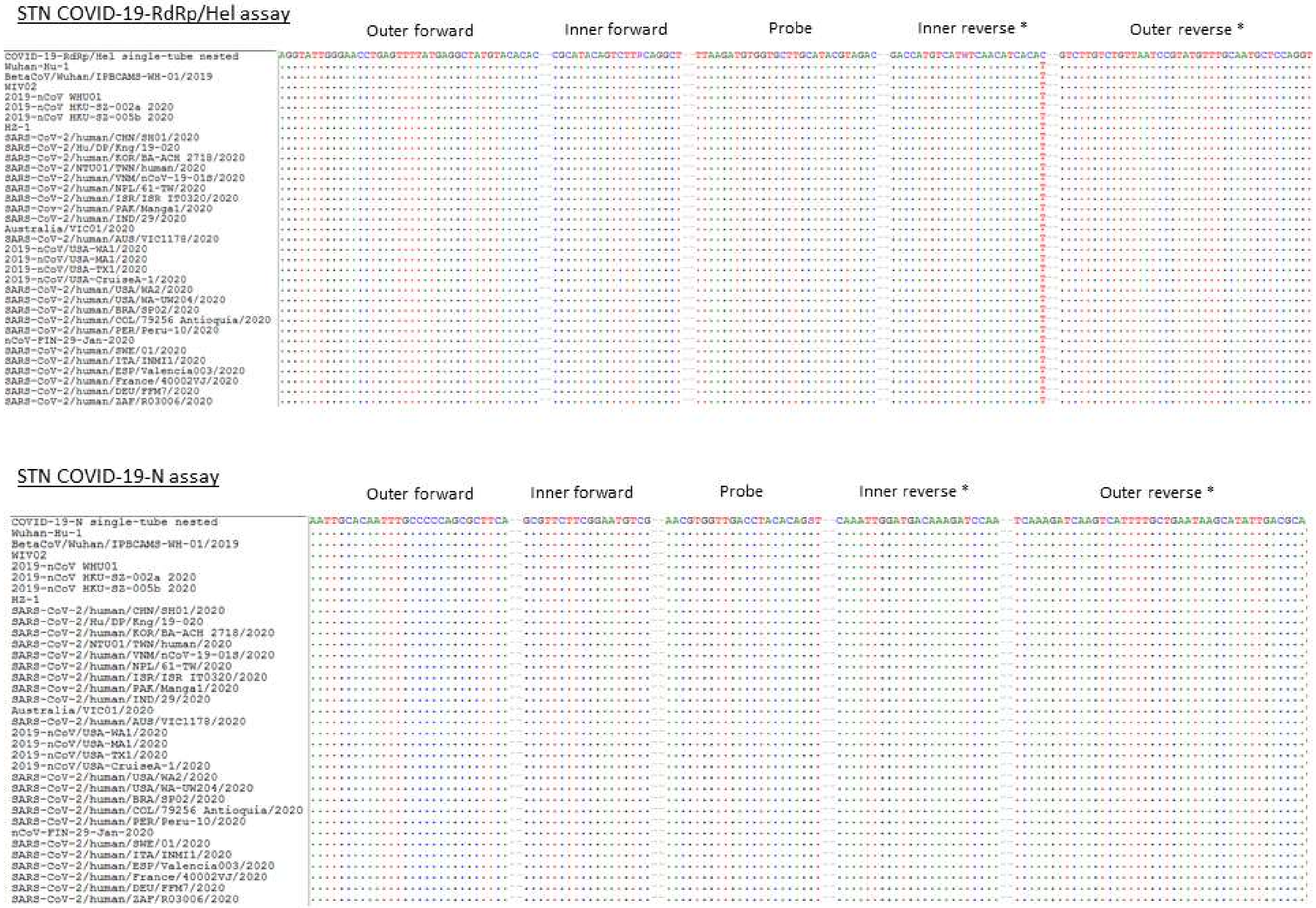

Figure S1. Multiple sequence alignments using the primer/probe sequences of the STN assays and the gene sequences of global SARS-CoV-2 isolates. *reverse-complement of reverse primer sequence

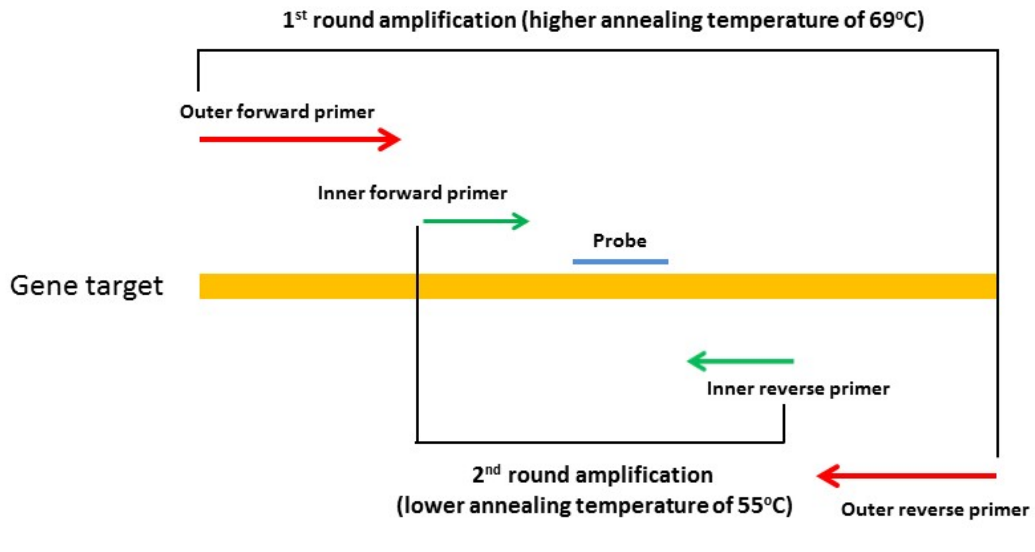

Figure S2. A schematic diagram of the in-house STN assay for the detection of SARS-CoV-2
